# Advances in application of single-cell RNA sequencing in cardiovascular research

**DOI:** 10.3389/fcvm.2022.905151

**Published:** 2022-07-26

**Authors:** Yue Hu, Ying Zhang, Yutong Liu, Yan Gao, Tiantian San, Xiaoying Li, Sensen Song, Binglong Yan, Zhuo Zhao

**Affiliations:** ^1^Department of Cardiology, Jinan Central Hospital, Shandong University, Jinan, China; ^2^Department of Cardiology, Central Hospital Affiliated Shandong First Medical University, Jinan, China; ^3^Department of Research Center of Translational Medicine, Central Hospital Affiliated Shandong First Medical University, Jinan, China; ^4^Department of Emergency, Central Hospital Affiliated Shandong First Medical University, Jinan, China

**Keywords:** single-cell RNA sequencing, cardiovascular, heart development, stem cells, precision medicine

## Abstract

Single-cell RNA sequencing (scRNA-seq) provides high-resolution information on transcriptomic changes at the single-cell level, which is of great significance for distinguishing cell subtypes, identifying stem cell differentiation processes, and identifying targets for disease treatment. In recent years, emerging single-cell RNA sequencing technologies have been used to make breakthroughs regarding decoding developmental trajectories, phenotypic transitions, and cellular interactions in the cardiovascular system, providing new insights into cardiovascular disease. This paper reviews the technical processes of single-cell RNA sequencing and the latest progress based on single-cell RNA sequencing in the field of cardiovascular system research, compares single-cell RNA sequencing with other single-cell technologies, and summarizes the extended applications and advantages and disadvantages of single-cell RNA sequencing. Finally, the prospects for applying single-cell RNA sequencing in the field of cardiovascular research are discussed.

## Introduction

Cardiovascular disease is considered to be the leading cause of human mortality worldwide. The latest data show that cardiovascular disease morbidity and mortality are gradually increasing (1). The pathogenesis related to the cardiovascular system has not been fully elucidated due to the diverse cellular interactions and complex regulatory mechanisms. Although there has been great progress in the diagnosis and treatment of cardiovascular disease owing to the development of anatomy, genomics, and other disciplines and the development of inspection technology (2), understanding the cellular heterogeneity and gene interactions in cardiac tissue development and cardiac cell differentiation trajectories and in disease states requires further research. The limitations of action hinder the in-depth study of the pathogenesis and the effectiveness of diagnosis and treatment plans. Therefore, how to use more powerful tools to research cardiovascular diseases and thereby promote cardiovascular health is still an important problem for researchers and clinicians to solve.

To achieve the goal of accurate disease prevention, diagnosis, and treatment, precision medicine not only involves interpreting big data about the living environment and habits of people but also strives to develop the fields of genomics and proteomics to reveal key differences related to the occurrence and development of diseases. The emergence and rapid development of single-cell technologies and omics analyses have facilitated a major leap in cardiovascular precision medicine. With the completion of the Human Genome Project (3), single-cell sequencing technologies have developed rapidly and have brought about progress in experimental biological research. Among the technologies, single-cell RNA sequencing (scRNA-seq) is of great significance in cardiac precision medicine. It captures the transcriptional profiles of thousands of single cells from complex tissues in high resolution. It thereby helps researchers to identify and target abnormal pathways and genes in cardiovascular disease and also assists clinicians in formulating effective treatment strategies (4). The research application of scRNA-seq is very extensive. In addition to revealing cardiovascular cell diversity, identifying stem cell differentiation trajectories, and clarifying cell-to-cell communication (5, 6), scRNA-seq has been used to characterize cardiac development and genetics (7, 8), which has promoted advances in cardiovascular research. This article reviews the application of scRNA-seq in relation to the cardiovascular system and provides an overview of future research strategies and directions.

## Single-cell transcriptome sequencing

**Figure 1:**
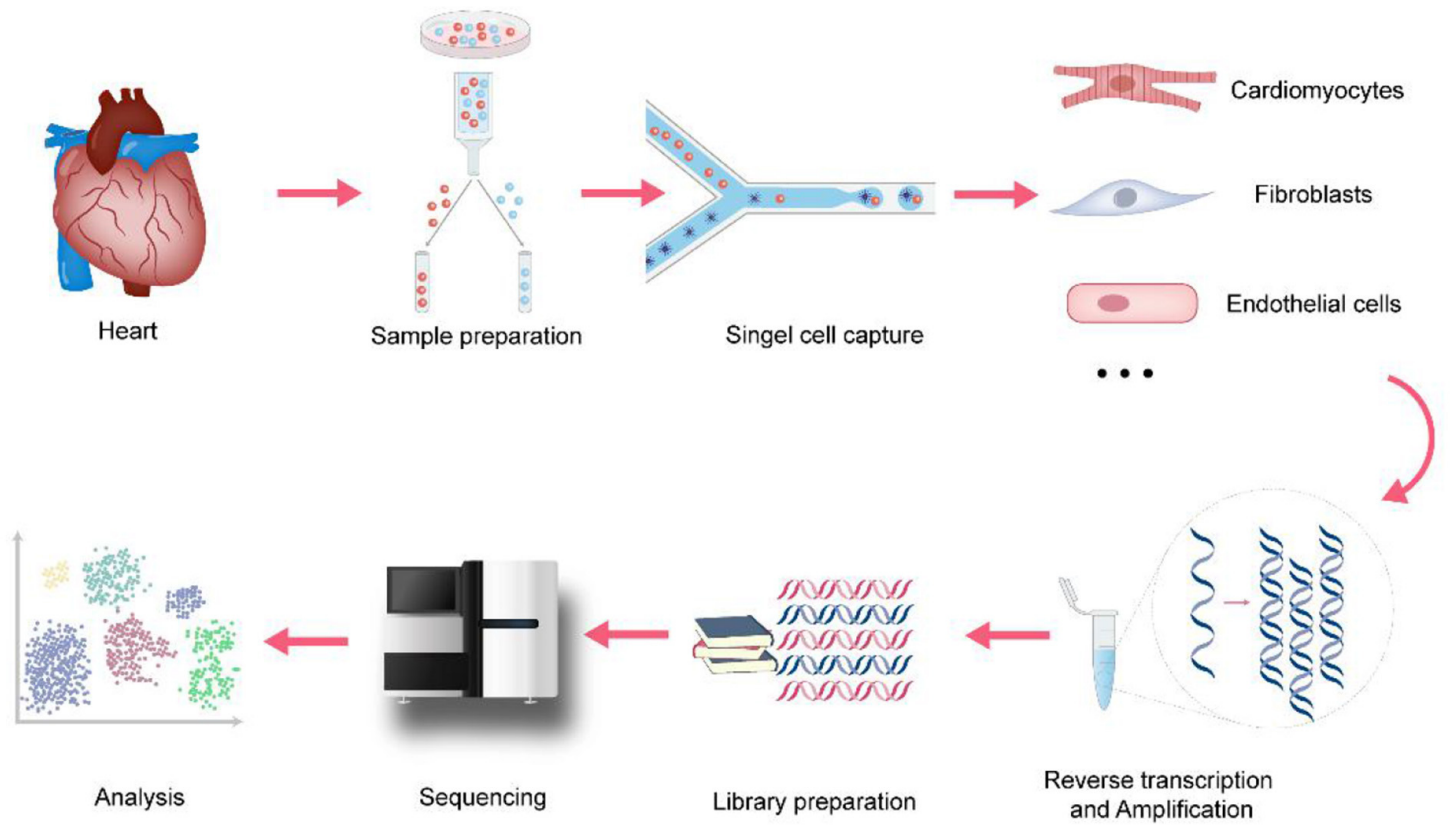
The process of single-cell RNA sequencing in cardiovascular research. In cardiovascular research, the basic scRNA-seq process involves: (1) sample preparation, Langendorff method and Enzymatic bulk digestion are two major methods for isolation of cardiomyocytes (2) single-cell capture, snRNA-seq holds advantages when applied to heart tissues because of the size of cardiomyocytes (3) amplification and library preparation, amplification methods are divided into two categories: single-cell whole-genome amplification (WGA) and single-cell whole transcriptome amplification (WTA) (4) sequencing, it involves single-cell genome sequencing, or single-cell transcriptome sequencing and single-cell epigenetic sequencing (5) analysis, cell clustering analysis, Differential expression analysis, Pseudotime analysis, Gene Ontology analysis and RNA velocity are the major analysis methods.

Since Tang et al. established the initial scRNA-seq process in 2009 (9), a variety of single-cell transcriptomics platforms have emerged one after another, with different degrees of optimization regarding single-cell capture methods, amplification methods, and transcript coverage. The development and improvement of scRNA-seq technologies will help to fully understand the changes at the gene expression level in both physiological and pathological conditions, thereby improving the precision of disease diagnosis and treatment. As shown in Figure 1, the basic scRNA-seq process involves: (1) sample preparation, (2) single-cell capture, (3) amplification and library preparation, and (4) sequencing and analysis (10).

### Sample preparation

Single-cell sequencing is based on the use of mechanical dissociation and enzymatic digestion to isolate single-cell samples from tissues. Different tissues or cell types, cell culture conditions, and cell viability require different tissue processing and digestion methods. For dense tissues such as the heart, the maximum number of cardiomyocytes is usually obtained by a combination of sectioning and enzymatic digestion (11). Langendorff method and Enzymatic bulk digestion are two major methods for isolation of cardiomyocytes. Enzyme digestion of heart tissue usually does not yield a sufficient number of viable cells due to insufficient tissue exposure (12). Langendorff method depends on tissue structure and has a complex operation process (13). So, considering the limitations of the two methods, researchers have made efforts to isolate single-cell samples from heart tissues. Based on Langendorff Method, Ackers-Johnson et al. (14) presented a novel method to isolate viable cardiac myocytes and non-myocytes from the adult mouse heart with only standard surgical tools and equipment, without the prerequisite of heparinization. Similarly, Guo et al. (11) developed a simplified method to isolate human cardiomyocytes from tissue slicing. It involves three steps: slice the tissue, Ca2^+^-free perfusion for 9 min, and enzymatic digestion for 45–90 min. This method can not only ensure the survival rate of cardiomyocytes, but also the integrity of morphology and morbid metabolic characteristics, the physiological function of cardiomyocytes. For soft tissues such as lymph nodes, single-cell suspensions can be obtained directly by mechanical separation (15). Researchers should optimize sample preparation procedures to maximize the number of single cells and viability while minimizing cell mortality.

### Single-cell capture

The single-cell capture method varies with the cell volume. Mainstream single-cell capture methods include laser capture, fluorescence-activated cell sorting (FACS), microfluidic capture, and microdroplet-based capture (16–18). FACS uses the principle of light scattering and fluorescence signals to sort cells into 96- or 384-well plates. Cell samples can be stored for a long time, increasing the flexibility of the research on the cells and reducing damage to the cells, but the plate-based limitations of FACS restrict the number of cells that can be sorted. The Fluidigm C1 system is one of the main technologies that use microfluidic cell capture. Its advantages are that it uses a smaller volume of cell suspension and reduces the risk of external contamination of cells (10). However, it requires a large number of live cells and cell size uniformity is limited in terms of the scale of analysis, and is expensive (10). Microdroplet-based platforms such as Drop-seq and Chromium (10x Genomics), which use DNA barcoding technology to analyze individual cells encapsulated in oil droplets, can rapidly analyze thousands of individual cells, greatly reducing the amount of time required for each analysis and cost. However, it still has the disadvantages of a low mRNA capture rate and low gene detection efficiency of cardiomyocytes due to their large cell size (4). However, Seq-wall and SPLiT-seq can effectively solve the problem. In Seq-wall, barcoded mRNA capture beads and single cells are sealed in an array of subnanoliter wells using a semipermeable membrane, enabling efficient cell lysis and transcript capture (19). So this technology is applicable to almost any cellular suspension for which a reference transcriptome exists. Similarly, In SPLiT-seq, individual transcriptomes are uniquely labeled by passing a suspension of formaldehyde-fixed cells or nuclei through four rounds of combinatorial barcoding. It eliminated the need for single-cell isolation because of the index information of DNA barcodes (20). These two methods have unique technical advantages to achieve a high mRNA capture rate of cardiomyocytes and are inexpensive, portable, and efficient. In addition, researchers have widely used Single-nucleus RNA-seq (snRNA-seq) in cardiovascular research (21). snRNA-seq holds several advantages when applied to heart tissues. First, it can be used to study frozen and archived primary tissues without the preparation of single-cell suspension. Second, it will minimize the bias of cell capture of platform-specific that has a certain size optimal and reduce the impact of aberrant transcriptional changes induced by enzymatic dissociation.

### Amplification and library preparation

Single-cell sequencing technology avoids the shortcomings of traditional technology that takes cell populations as the research object and ignores the heterogeneity between cells. Using a single cell as the research object, single-cell sequencing technology lyses a single cell and can amplify ultra-trace amounts hundreds of thousands of times, and establishes a sequencing-level cDNA library for sequencing and data analysis (22).

To avoid amplification bias, researchers should choose the most optimized method based on sensitivity, precision, and accuracy. The commonly used amplification methods are mainly divided into two categories: single-cell whole-genome amplification (WGA) and single-cell whole transcriptome amplification (WTA). The classification of these methods is shown in Table 1. The difference between WTA and WGA is that WTA first reverse-transcribes RNA into cDNA, and then amplifies the cDNA. Among the types of WTA, CEL-seq uses a unique molecular identifier (UMI) in the cDNA synthesis process and linearly amplifies mRNA, thereby reducing amplification bias (32), and it is more cost-effective for transcriptome quantification of a large range of expression levels. Another type of WTA, Smart-seq2, uses Moloney murine leukemia virus (MMLV) reverse transcriptase, which preferentially selects full-length cDNA as a substrate for its terminal transferase activity, allowing the recovery of full-length cDNAs. It thereby improves the reverse transcription process and increases the yield and length of single-cell cDNA libraries. It achieves high sensitivity and low amplification bias. Additionally, it can analyze all exons of each single-cell transcript, detect different splice variants, and has a wider range of applications (30).

**Table 1:** Comparison of single-cell sequencing technology gene amplification methods.

**Amplification type**	**Amplification method**	**Advantages**	**Limitations**	**References**
WGA	DOP-PCR	CNV detection in a larger genome.	Low genome coverage and high error rate for SNV detection	(23)
	MDA	high genome coverage	Large bias and susceptible to contamination	(23)
	MLBAC	Small sequence-dependent bias, high CNV detection accuracy, and low SNV false negative rate	Low fidelity and high SNV false positive rate	(24)
	eWGA	Good amplification uniformity, strong sensitivity for both CNV and SNV, and low contamination rate	To be studied	(25)
	LIANTI	High gene coverage, low allele loss, and good amplification uniformity	Less accurate for very small CNVs	(26)
	SISSOR	High sequencing accuracy for undivided cells	to be studied	(27)
	PicoPLEX	Low amplification error rate, sensitive for CNV, good repeatability and amplification uniformity	To be studied	(28)
WTA	CEL-seq	High reproducibility and sensitivity and short amplification times	Low Specificity For mRNA amplification	(29)
	Smart-seq/Smart-seq2	Low amplification bias, high coverage, low variability, and low noise	No analytical ability for polyA ribonucleic acid	(30)
	Drop-seq	Low cost and fast amplification	Low mRNA capture rate	(31)

*WGA, single cell whole gene group amplification; WTA, single-cell transcriptome amplification; CNV, copy number variation; SNV, single-nucleotide variation; DOP-PCR, degenerate oligonucleotide primed PCR; MDA, multiple strand displacement amplification; MLBAC, multiple annealing circular cyclic amplification; eWGA, emulsion whole genome amplification; LIANTI, transposon insertion linear amplification; SISSOR, microfluidic reactor single strand sequencing; CEL-seq, Cell Expression by Linear amplification and Sequencing; Smart-seq, Switching mechanism at 5' end of the RNA transcript*.

### Sequencing and analysis

At present, the third-generation single-cell transcriptome sequencing technology has been developed. The representative technologies are HeliScope single-molecule sequencing (33), single-molecule real-time sequencing (34), Oxford nanopore sequencing (35). Compared with the second-generation sequencing technology, the third-generation sequencing technology uses useful information such as nanotechnology and modern optics to capture the base sequence, which has the advantages of fast, real-time sequencing and longer base sequence reading. However, there is still room for improvement in the cost and accuracy of sequencing (36).

In addition to the single-cell genome or transcriptome sequencing, single-cell sequencing methods also include single-cell epigenetic sequencing. The innovation regarding single-cell epigenetic sequencing is that the methylation level of the whole genome can be obtained, which is of great significance for the study of epigenetics as it allows the spatiotemporal specificity of epigenetic changes to be determined.

Currently, multi-omics sequencing technologies (37, 38), including G&T-seq (39), TARGET-seq (40), scCAT-seq (41), scM&T-seq (42), and PLAYR (43) technologies, are booming. Compared to single-omics sequencing technologies, multi-omics sequencing technologies can provide more systematic insights into understanding biological heterogeneity and diversity. Multi-omics sequencing analysis that involves both spatial data and time trajectory data avoids some of the shortcomings of single-omics sequencing and enriches the experimental results. Multi-omics sequencing will certainly be a potential method in the field of sequencing research in the future.

Regarding the analysis of single-cell sequencing data, cell clustering analysis is a key type of data analysis. It is the basis of further analysis of the data (44), such as differential expression analysis, pseudotime analysis, and Gene Ontology analysis. And the downstream analysis such as RNA velocity is also important. A comparison of these methods is shown in Table 2. In addition, the multiplexing method increases the number of samples for scRNA-seq, facilitates library construction, and lowers the reagent costs by relying on DNA-based barcoding that enables the pooling of all barcoded samples into a single mixed sample for analysis. It will provide great insight into high-throughput perturbation screening and tracking the dynamic process of cell differentiation (55). Researchers need to select a data analysis method according to their research purposes and conditions.

**Table 2:** Comparison of single-cell sequencing data analysis methods.

	**Principles of data analysis**	**Core algorithm**	**Advantage**	**References**
Differential expression analysis	Aggregation analysis in which the effects of different samples or treatment methods on gene expression levels are compared	SCDE, MAST Census, BCseq	Identifies cell-specific markers and distinguishes among various cell subsets	(45–47)
Pseudotime analysis	Dynamic pathways of cell development or differentiation are inferred based on gene expression patterns in single cells	Monocle	Identifies key genes in cell differentiation in a non-purified state	(48–51)
Gene Ontology analysis	Controlled word sets, which comprehensively describe the properties of genes and gene products, are identified	Gene Ontology	Determines accurate descriptions of cells and molecular functions	(52)
Cell clustering analysis	Define cell types through unsupervised clustering on the basis of transcriptome similarity	k-means	Identifies putative cell types in any samples	(53)
RNA velocity	Recovers directed information by distinguishing unspliced and spliced mRNAs	velocyto, scVelo	Grants access to the descriptive state of a cell, and its direction and speed of movement in transcriptome space	(54)

## Application of single-cell transcriptome sequencing in the cardiovascular system

### Cellular heterogeneity in the developing and mature heart

With the development of scRNA-seq, it has become possible to reveal new cell types and cell subpopulation heterogeneity in the heart, and to describe the spatial and temporal expression patterns of different cell types. For example, by performing scRNA-seq on NKX2.5^+^ and Isl1^+^ mouse cardiac progenitor cells (CPCs) and developmental trajectory analysis, Jia et al. (56) revealed that Isl1^+^ CPCs undergo an attractor state before entering various developmental branches, while extended expression of NKX2.5 may promote the unidirectional development of CPCs into cardiomyocytes. This demonstrated that Nkx2.5 expression plays a decisive role in the differentiation of pluripotent CPCs into cardiomyocytes. Similarly, Xiao et al. (57) performed scRNA-seq on mouse embryonic hearts and found that *Lats1* and *Lats2* (in the Hippo signaling pathway) caused subepicardial cells to differentiate into cardiac fibroblasts by inhibiting the YAP target gene *Dhrs3*. After conditional knockout of *Lats1* and *Lats2*, aberrant signaling from C20, a transitional cell subset between the epicardium and cardiac fibroblasts, led to a disordered coronary configuration. *Lats1* and *Lats2* also play important roles in controlling extracellular matrix composition and vascular remodeling while promoting the transition of epicardial progenitors to differentiated cardiac fibroblasts. Moreover, by performing scRNA-seq on cardiac differentiation from human embryonic stem cells and human embryonic/fetal hearts. Sahara et al. (58) identified a unique cell subset that emerges specifically in the proximal outflow tract of human embryonic hearts, marked by LGR5. And the LGR5^+^ cells promote cardiogenesis through expansion of the ISL1^+^TNNT2^+^ intermediates. This study will help to gain insight into human cardiac development and reveal the possible origin of congenital heart disease. Thus, scRNA-seq is important for characterizing the heterogeneity of developing and mature cardiac cells and for identifying the molecular mechanisms of cardiac development by allowing individual cardiac cells to be studied.

### Induced pluripotent stem cells

iPSCs have been widely used in research on the molecular mechanisms of cardiovascular-related diseases. However, the endothelial cells and cardiomyocytes derived from iPSCs have differences in physiological structure and gene expression compared to mature cardiac cells, which is a barrier to the application of iPSCs in clinical treatment (59). scRNA-seq can capture the regulatory processes of stem cell differentiation and development with unprecedented sensitivity, providing technical support and a theoretical basis for stem cell transplantation (60). Paik et al. (61) performed large-scale droplet-based scRNA-seq on thousands of human iPSC-derived endothelial cells. They found that *CLDN5, APLNR, GJA5*, and *ESM1* enrichment characterized four cell subsets, and they elucidated the unique physiological role of each cell subset, providing a foundation for sorting IPSC with specific biological function and identity. In addition, by performing scRNA-seq on human iPSC-derived cardiomyocytes, Churko et al. (62) revealed that *NR2F2* and *HEY2* promote atrial and ventricular-like effects, respectively. The fact that these genes have different regulatory roles that lead to different functional cell states was validated in mouse models (63). They also found that notch signaling may be regulating the atrial vs. ventricular development within different heart chambers. These findings will help to promote the clinical translation of iPSCs research into cardiovascular disease treatments.

### Cardiovascular disease

#### Congenital heart disease

Compared to traditional sequencing technology, scRNA-seq provides more insight into congenital heart disease caused by genetic changes at the single-cell level. By studying autoimmune congenital heart block (CHB) fetal hearts and healthy fetal hearts, Suryawanshi et al. (64) revealed that overexpression of interferon-stimulated genes and activation of interferon signaling were involved in the occurrence of CHB. They also found that stromal cells may contribute to stromal deposition and fibrosis, leading to CHB. Likewise, by using scRNA-seq to study mouse cardiac progenitors, de Soysa et al. (65) demonstrated that *Hand2* is a transcriptional determinant of individual CPC fate and differentiation direction. They found that *Hand2-*knockout mice have abnormal right ventricle formation due to defective ventricular outflow tract development, resulting in congenital heart disease phenotypes. In addition, scRNA-seq also provides a higher discriminative power regarding genetic alterations in specific cell types that trigger congenital heart disease. Hu et al. (21) compared the single-cell transcriptome sequencing results of nuclei from the hearts of mice with mitochondrial cardiomyopathy and normal mouse hearts. They found that the growth factor *GDF15* was up-regulated in the former, revealing the cardiac cell type-specific gene regulatory network of *GDF15*. These studies demonstrate that the application of scRNA-seq not only paints a new picture of cardiac development from a completely new perspective, but also provides new information on genes and mechanisms for further elucidation of congenital heart disease.

#### Heart disease in adults

There is a growing interest in conducting research on cardiac disease pathogenesis and novel therapeutic targets, and scRNA-seq is revolutionizing our understanding of common cardiac diseases. Using a pressure overload-induced heart failure model, scRNA-seq provides insights into cardiac cellular heterogeneity, laying the foundation for therapeutic strategies for the disease. Nomura et al. (66) constructed trajectories related to cardiomyocyte remodeling by subjecting individual cardiomyocytes to scRNA-seq, elucidating the genetic programs underlying the morphological and functional characteristics of cardiac hypertrophy and failure. In the early stage of cardiac hypertrophy, cardiomyocytes activate mitochondrial metabolic genes, which are related to cardiomyocyte size and connect to the *ERK1/2* and *NRF1/2* transcriptional networks to initiate myocardial remodeling. In the late stage of cardiac hypertrophy, the p53 signaling pathway is activated, followed by cardiomyocyte morphological elongation and heart failure. Furthermore, scRNA-seq has been used to track cell fate transitions in the early, developing, and convalescent phases of myocardial infarction, providing new insights into the pathogenesis of myocardial infarction (67). In the early phases of myocardial infarction, fibroblasts are activated, creating a collagen scar that reduces further cardiac tissue rupture. But in the convalescent phases of myocardial infarction, fibroblasts will eventually lead to heart failure (68). Therefore, studying activated fibroblasts helps us to better understand their role in the pathogenesis of myocardial infarction. By performing scRNA-seq on collagen 1α1 green fluorescent protein (GFP)^+^ fibroblasts after myocardial infarction, Ruiz-Villalba et al. (69) found that activated fibroblasts up-regulate collagen triple helix repeat containing 1 (*Cthrc1*) in the early stage of myocardial infarction and participate in collagen synthesis through TGF-β signaling, to promote healing and prevent heart rupture. *Cthrc1* was down-regulated during the myocardial infarction recovery period to avoid pathological ventricular remodeling.

#### Vascular lesions

Atherosclerosis (AS) is a chronic inflammatory disease associated with vascular endothelial cell dysfunction, myeloid cell enrichment, lipid deposition and foam cell formation (70–72). scRNA-seq has advanced the understanding of AS as a multifactorial disease involving diverse cellular interactions (see Table 3 for details). In addition, the application of scRNA-seq to abdominal aortic aneurysm (AAA) research and the development of spatial transcriptomics have led to a deeper understanding of the cellular heterogeneity involved in AAA and the functional status of the various cells. Yang et al. (79) performed scRNA-seq (based on the 10x Genomics) platform on aortic cells in the early stage of AAA. They observed the number of macrophages was increased. And Mϕ-2, an inflammatory macrophage, was found to be the main enriched macrophage type in the early stage of the disease. Thus, scRNA-seq revealed the types of smooth muscle cells and macrophages involved in the development of AAA, and predicted the functional status of cells, in order to improve research on AAA pathogenesis, targeted drug development, and personalized vascular medicine.

**Table 3:** Research on single-cell sequencing of aortic cells in atherosclerosis (AS).

**Species**	**Cell type**	**Platform**	**Main findings**	**References**
Mouse	CD45^+^ leukocytes	Fluidigm C1	Depiction of immune cell composition and transformation trends within atherosclerotic arterial vessels	(73)
Mouse	Th1-like IFNγ^+^CCR5^+^ Treg subset (Th1/Tregs), T regulatory (Treg) cells, and Th1 cells	Fluidigm C1	AS involves Treg plasticity, accumulation of interferon gamma^+^ Th1/Tregs, Treg subpopulation dysfunction, and further promotes arterial inflammation	(74)
Mouse/human	Smooth muscle cells	10x Genomics	TCF21 regulates the transition of smooth muscle cells to fibromuscular cells in AS, and the latter protects against AS by infiltrating lesions	(75)
Mouse	Adventitial cells	10x Genomics	Descriptive cellular atlas of heterogeneous cell populations in the adventitia, revealing dynamic interactions between adventitial macrophages and stroma in AS	(76)
Mouse	Macrophages	10x Genomics	Non-foaming macrophages promote inflammation in AS	(77)
Human	smooth muscle cells	ICELL8	Histone H2A variant H2A.Z was down-regulated in AS smooth muscle cells, and its overexpression inhibited VSMC dedifferentiation and neointima formation caused by injury, and played a protective role in AS	(78)

### Cardiac precision medicine

The purpose of precision medicine is to deliver individualized treatment in order to improve health. This involves constructing information repositories and infrastructure to improve the efficiency of clinical research, and to provide more precise information in clinical settings (80). In cardiovascular system applications, scRNA-seq has enabled the identification of disease-causing gene expression and mutations at the single-cell level, and it has provided unprecedented insights into cell developmental trajectories and differentiation directions. Hulin et al. (81) used droplet-based transcriptome sequencing to show that the gene expression and function of endothelial and immune cell subsets remained relatively constant during aortic and mitral valve development in neonatal d7 (primitive values) and d30 (mature valves) mice, while the interstitial cell subsets exhibited significant changes. This study was the first to reveal the cellular diversity during heart valve remodeling, opening up new avenues for studying heart valve homeostasis and the molecular mechanisms of valve diseases. Due to its unique technical advantages, scRNA-seq has played an important role in revealing cell-to-cell heterogeneity during disease development and drug treatment. Its role in identifying and thereby targeting complex cardiovascular pathological phenotypic pathways has also promoted the development of cardiac precision medicine, providing effective therapeutic strategies for cardiovascular diseases. However, scRNA-seq still has shortcomings such as excessive raw data noise and low gene coverage (82). It is believed that with continuous scRNA-seq innovations related to the current technical barriers, its contribution to cardiac precision medicine will become more significant.

### Coronavirus disease 2019 associated heart injury

Severe acute respiratory syndrome coronavirus 2 (SARS-CoV-2) binds to the ACE2 receptor and transmembrane protease serine 2 (TMPRSS2) cleaves the viral S protein (83). This activates the process of entry into host cells to allow the virus to infect these cells, which eventually leads to novel coronavirus-associated pneumonia (COVID-19) and cardiac damage (84). ACE2 and TMPRSS2 double positivity is a key condition for SARS-CoV-2 to enter cells. Liu et al. (85) compared the scRNA-seq data of fetal and adult human heart tissue with lung tissue data and showed that ACE2 was relatively up-regulated in heart tissue compared to lung tissue, but due to the low TMPRSS2 expression, the heart damage was minor. Thus, a lower percentage of ACE2^+^ TMPRSS2^+^ cells somewhat reduce susceptibility to SARS-CoV-2-induced cardiac damage. Nevertheless, up-regulated cathepsin L (CTSL) and paired basic amino acid-cleaving enzyme (furin) in the heart may compensate for TMPRSS2, mediating SARS-CoV-2 infection of the heart (85). Sequencing single adult coronary artery cells showed that compared to endothelial cells from the lung, ACE2 was up-regulated in cardiac endothelial cells, but TMPRSS2 was down-regulated, so the endothelial cells may act as a barrier that protects the cardiac tissue from circulating SARS-CoV-2 (85). The above studies show that single-cell sequencing, as a powerful technical means, can help to further understand the cardiovascular damage caused by SARS-CoV-2 and its underlying mechanisms, thereby promoting the identification of treatment targets and reducing mortality.

## Expansion of the application of scRNA-seq

### Cell–cell interactions

Recognition and signal transmission between cells is carried out through receptor-ligand binding. Accurate CCIs based on this process are an important prerequisite for organisms to maintain complex life activities (86). Therefore, the study of the CCI process is beneficial to advancing the interpretation of cell biological functions, metabolic states, disease pathological processes, and other aspects.

Deciphering CCIs based on gene expression using scRNA-seq has been greatly developed. Compared to traditional analytical methods, it has greater advantages in quantifying gene expression in rare cell types and identifying cellular sources of proteins that mediate CCIs. For example, Skelly et al. (87) constructed a framework of the extensive networks of intercellular communication in the heart by performing scRNA-seq on mouse non-cardiomyocyte cells. Similarly, Wang et al. (88) compared the interactions of human cardiomyocyte cells and non-cardiomyocytes in healthy and failing states based on scRNA-seq, which revealed the regulation of cardiomyocyte behavior by non-cardiomyocyte cells.

### Trajectory analysis

Traditional lineage tracing methods involve tracking the expression levels of specific genetic markers in progeny cells (89). Due to the limited number of genetic marker genes available, it is impossible to accurately track all progeny cell types, so information on cell heterogeneity is incomplete. However, with the development of scRNA-seq technologies and computational methods, trajectory analysis based on scRNA-seq data makes it possible to trace cell development and differentiation lineages without using clear genetic markers (90). Among the methods, pseudo-time trajectory analysis based on the Monocle algorithm is the most representative (91). The principle is to infer the trajectory based on the similarity of expression patterns between the sequenced cells, and then to sort the single cells in one-dimensional space to indicate the trajectory. In this way, information on the complex dynamic differentiation process of cells can be obtained (92).

However, pseudo-time trajectory analysis cannot completely replace the traditional lineage tracing methods due to the disadvantages of non-linear intermediate product interference, unstable sequencing depth, and obvious batch effects. Nevertheless, the calculation methods involved in pseudo-time trajectory analysis are still being continuously improved. Currently, Monocle3, which is an improved version of Monocle, is being used for trajectory analysis of limbal basal epithelial cells (93) and analysis of tumor cell genetic mutations (94). With the continuous advancement of technology, it is expected that developmental trajectory analysis will be combined with spatial transcriptomics analysis and data on cell and molecular properties. These methods will then play greater roles in revealing the gene expression signatures of different cells and developmental stages and in constructing embryonic developmental maps of unexplored species.

## Comparison with other single-cell technologies

### Single-nucleus RNA sequencing

Compared to scRNA-seq, snRNA-seq has the advantages of less tissue dissociation bias, high compatibility with frozen samples, and elimination of tissue dissociation-induced transcriptional stress responses because it is not limited by the dissociation conditions (95). It has been widely used in many organs including the heart. By performing snRNA-seq on adult mammalian hearts, Wolfien et al. (96) found that mature cardiomyocytes are not of a single origin and identified a cardiomyocyte subpopulation of endothelium-oriented origin with dual roles. Likewise, Galow et al. (97) found that the turnover of the cardiomyocyte pool is generated by cytokinesis of resident cardiomyocytes rather than being driven by the differentiation of progenitor cells. These studies contribute to the understanding of cardiac cell biology, including cardiomyocyte regeneration. In addition, by performing snRNA-seq on left ventricle samples from hearts with dilated cardiomyopathy and hypertrophic cardiomyopathy as well as non-failing hearts, Chaffin et al. (98) found that in cardiomyopathic hearts, the expression of proliferating resident cardiac macrophages was reduced and that of activated fibroblasts was increased. *PRELP* and *COL22A1* played a plausible role in cardiac fibrosis by encoding the extracellular matrix protein prolargin in fibroblasts. Their research will expand our understanding of the transcriptional and molecular basis of cardiomyopathy.

However, snRNA-seq has strict nuclear extraction conditions and complex procedures. In addition, the level of mRNA in the nucleus is relatively low, it is estimated that only 50% of RNA is present in the nucleus vs. cytoplasm, so snRNA-seq is not highly sensitive for all cells and genes (99). Thrupp et al. (100) compared the performance of snRNA-seq and scRNA-seq for analyzing human cortical microglia and found that snRNA-seq was less sensitive at identifying some genes (such as *APOE* and *CST3*) related to Alzheimer's disease, showing that snRNA-seq provides insufficient insights into some genes in some human tissues and cells. Additionally, Slyper et al. (99) showed that snRNA-seq was less powerful than scRNA-seq for the analysis of immune cells in fresh and frozen human tumor samples. Therefore, researchers should combine snRNA-seq data with cell phenotyping and proteomics to gain accurate and comprehensive information.

In addition, the combination of snRNA-seq and single nucleus assay for transposase-accessible chromatin sequencing (snATAC-seq) has been used in experimental research. By analyzing transcriptomic and epigenomic interactive multimodal atlas by Combining snRNA-seq and snATAC-seq, Muto et al. (101) highlighted functional heterogeneity in the proximal tubule and thick ascending limb. Similarly, Thomas et al. (102) identified transcription-factor-binding motifs and cis-regulatory elements in the human retina and induced pluripotent stem cell-derived retinal organoids through the use of snATAC-seq and snRNA-seq. This combination will provide a unique opportunity to utilize tissues that have been already obtained and help researchers get more information.

### Mass cytometry (cytometry by time-of-flight mass spectrometry)

As post-translational modification is a key process in gene regulation and signaling pathway activation, there is a lack of one-to-one correspondence between mRNA and protein levels in cells. Therefore, the application of single-cell technologies in the field of proteomics helps to gain accurate information on disease pathogenesis. Among the technologies, mass cytometry (CyTOF) is a multi-parameter single-cell technology. Owing to its high accuracy in single-cell analysis and wide range of measurement parameters, it can accurately analyze intracellular signaling networks and immunophenotypes, making it a powerful single-cell proteomics tool (103). For example, by performing CyTOF and scRNA-seq on carotid plaques and T cells of AS patients, Fernandez et al. (104) found that CD4^+^ T cell subsets differed between AS patients and healthy individuals, with some T cell subsets showing signs of exhaustion. Identification of macrophage subsets associated with carotid plaque vulnerability has furthered research on cardiovascular immunotherapies (104). Similarly, Chen et al. (105) performed CyTOF on the aortic smooth muscle cells of a mouse model of the aneurysm and found that *KLF4* reduces TGF-β signaling and reprograms contractile smooth muscle cells into mesenchymal stem cells, thus promoting the generation and progression of aneurysms.

### “Spatial transcriptomics” technologies

scRNA-seq has unique advantages regarding providing information on cellular heterogeneity and disease pathogenesis, but there are still some limitations. The extraction of single cells precludes collecting detailed information on their specific location in tissues and this makes scRNA-seq less effective for functional interpretation of gene expression in specific physiological or pathological microenvironments (106). To obtain both cellular location and gene expression data, in 2016 Ståhl et al. (107) developed spatial transcriptomics technologies to capture mRNAs along with location data in tissues (using unique location barcodes) in order to visualize gene expression distribution in tissues.

In recent years, the development of spatial transcriptomics technologies has been rapid. Among them, tissue-based labeling technology represents the latest technological progress in spatial transcriptomics. The ZipSeq technology invented by Hu et al. (108) is a representative living tissue labeling technology. It uses a light control system to map DNA barcodes to living cells to identify real-time gene expression levels in specific cells. It is beneficial to combine spatial information with surface epitope profiling, and thereby promote understanding of the interconnections between cellular locations and transcriptional heterogeneity. Spatial Transcriptomics frequently provides technical support for research on tissue and cell spatial heterogeneity and gene expression across many research fields such as developmental differentiation lineage mapping, cell functional state interpretation, and disease model exploration. It has become a core technical facility in the field of biological research.

### Omics analysis

Precision medicine will be improved by combining multi-omics approaches related to the epigenome, transcriptome, proteome, and metabolome. Epigenomics analysis, including the analysis of DNA methylation, histone acetylation, and phosphorylation, is an important component of the multi-omics analysis (109). Asare et al. (110) used bone marrow reconstitution experiments in mice with hyperlipidemia and epigenomics analysis to show that histone deacetylase 9 (HDAC9) binds to inhibitory kappa B kinase (IKK), resulting in IKK deacetylation and activation, and stimulating the inflammatory response of macrophages and endothelial cells. The research revealed that HDAC9 inhibition therapy delays AS progression.

Current research directions in the field of multi-omics involve reducing the experimental cost and combining more omics techniques together. In addition, metabolomic analysis of metabolic changes, which are located downstream of the genome—closer to the phenotype of the organism—can lead to a better assessment of the physiological changes that occur in diseased states (111). Since the appearance of metabolomic in 1999 (112), great progress has been made in understanding the pathological mechanisms underlying cardiovascular disease. For example, Murashige et al. (113) conducted a metabolomic study of arterial, coronary, and femoral venous blood from 110 heart failure and non-heart failure patients and found that fatty acids are the main energy source of the heart in both groups, but the hearts of the heart failure patients exhibited higher ketone and lactate consumption with a high proteolysis rate. The findings contribute to the construction of a framework describing human cardiac energy sources and provide a basis for understanding cardiometabolic abnormalities in disease states. Unlike scRNA-seq, metabolomics analysis can easily integrate upstream genetic data, transcriptional and proteomic variation, and cellular microenvironment information, so it can better reflect the molecular processes involved in disease states (114). At present, combined single-cell multi-omics analysis technologies are developing rapidly. Multi-omics technologies for analyzing the transcriptome, open chromatin, and histone modification has been gradually developed and has become an important tool for promoting the development of precision medicine (115).

## Discussion

The use of scRNA-seq in cardiovascular research has been greatly promoted by the innovation of single-cell capture methods, the development of gene amplification methods, the emergence of multiple sequencing platforms, and the diversification of data analysis methods. scRNA-seq has played an important role in exploring cardiac cell heterogeneity during heart development, improving stem cell models, and revealing the molecular mechanisms of diseases and potential therapeutic targets. In particular, it has been useful in research on the mechanisms of COVID-19-related cardiac injury, which may lead to rapid identification of therapeutic targets and reductions in mortality.

Combining single-cell sequencing with other technologies would also provide more useful information to researchers. Ranzoni et al. (116) applied scRNA-seq and scATAC-seq to human immunophenotypic blood cells from fetal liver and bone marrow, they identified transcriptional and functional differences between hematopoietic stem cells from liver and bone marrow. Similarly, Moncada et al. (117) performed microarray-based spatial transcriptomics with scRNA-Seq on pancreatic tumors, they identified cell type subpopulations across tissue regions and cell state relationships in the tumor microenvironment. In addition, Zhao et al. (118) applied pooled CRISPR delivery and single-cell transcriptome analysis to the βcell line MIN6, they confirmed that the lncRNA-enriched cluster of MIN6 was associated with insulin transcription.

However, scRNA-seq still has certain limitations. First, single-cell sorting platforms have limitations in capturing cardiomyocytes due to their large size. Second, compared to snRNA-seq, scRNA-seq has limitations such as low compatibility with frozen samples, inducing strong transcriptional stress responses that affect the results, and the inability to provide nuclear gene expression information. In addition, compared to CyTOF, scRNA-seq cannot take into account proteomics data alongside gene expression data, and it provides poor insights into pathological states caused by abnormal post-translational modification of genes. An enzyme-tethering strategy called Cleavage Under Targets and Tagmentation (CUT&Tag) has emerged (119). In CUT&Tag, a specific antibody binds to the target chromatin protein, which then tethers a protein A-Tn5 transposase. After activation of the transposase and DNA purification because of the addition of Mg^2+^, the genomic fragments with adapters at both ends are enriched by PCR. The steps from Sample preparation to amplification and library preparation can be performed in a single tube on the benchtop in 1 day. This strategy provides high-resolution and low background sequencing libraries for profiling diverse chromatin components and saves time. Therefore, there is still a lot of room for improvement of scRNA-seq technologies for use in cardiovascular system research. Future research should focus on developing more efficient platforms for cardiomyocyte capture, improving sequencing coverage, and reducing costs in order to conduct large-scale single-cell sequencing experiments to address clinical problems related to the cardiovascular system. For example, breakthroughs have already been made in the fields of metabolic heart diseases, including diabetic cardiomyopathy (120) and Alcoholic-dilated Cardiomyopathy (121). In addition, scRNA-seq should be improved in terms of sequencing technology standardization, and visualization of analysis results in order to meet the needs of integrated multi-omics research. It is believed that with continuous innovation, scRNA-seq will play a greater role in the field of cardiovascular system research and promote the development of precision medicine, including targeted diagnostic methods and treatments for cardiovascular diseases.

## Author contributions

YH collected the literature and wrote the manuscript with guidance from ZZ. YZ, YL, YG, TS, XL, SS, and BY were involved in making figures and tables. All the authors read and approved the manuscript and agreed to its publication.

## Funding

This work was supported by a grant from Natural Science Foundation of Shandong Province (No. ZR2021MH279 to ZZ).

## Conflict of interest

The authors declare that the research was conducted in the absence of any commercial or financial relationships that could be construed as a potential conflict of interest.

## Publisher's note

All claims expressed in this article are solely those of the authors and do not necessarily represent those of their affiliated organizations, or those of the publisher, the editors and the reviewers. Any product that may be evaluated in this article, or claim that may be made by its manufacturer, is not guaranteed or endorsed by the publisher.
